# A comparative analysis of the restaurant consumer food environment in Rochester (NY, USA) and London (ON, Canada): assessing children’s menus by neighbourhood socio-economic characteristics

**DOI:** 10.1017/S1368980018003804

**Published:** 2019-02-12

**Authors:** Catherine M DuBreck, Richard C Sadler, Godwin Arku, Jamie Seabrook, Jason Gilliland

**Affiliations:** 1 Department of Geography, University of Western Ontario, 1151 Richmond Street, London, Ontario, Canada, N6A 3K7; 2 Department of Family Medicine/Division of Public Health, College of Human Medicine, Michigan State University, Flint, MI, USA; 3 Children’s Health Research Institute, London, Ontario, Canada; 4 School of Food and Nutritional Sciences, Brescia University College, London, Ontario, Canada; 5 Department of Paediatrics, Department of Epidemiology & Biostatistics, School of Health Studies, University of Western Ontario, London, Ontario, Canada

**Keywords:** Food environment, Menus, Neighbourhood, Socio-economic status, Child health, Canada, USA

## Abstract

**Objective:**

To assess restaurant children’s menus for content and nutritional quality; and to investigate the relationship between the restaurant consumer food environment for children and neighbourhood-level socio-economic characteristics within and between one Canadian city and one US city.

**Design:**

Cross-sectional observational study.

**Setting:**

London, ON, Canada and Rochester, NY, USA.

**Participants:**

Restaurant children’s menus were assessed, scored and compared using the Children’s Menu Assessment tool. We quantified neighbourhood accessibility to restaurants by calculating 800 m road-network buffers around the centroid of each city census block and created a new Neighbourhood Restaurant Quality Index for Children (NRQI-C) comprising the sum of restaurant menu scores divided by the total number of restaurants within each area. After weighting by population, we examined associations between NRQI-C and neighbourhood socio-economic characteristics using correlations and multiple regression analyses.

**Results:**

Nutritional quality of children’s menus was greater, on average, in Rochester compared with London. Only one variable remained significant in the regression analyses for both cities: proportion of visible minorities had a positive effect on neighbourhood NRQI-C scores in London, whereas the reverse was true in Rochester.

**Conclusions:**

Results suggest the presence of a socio-economic disparity within Rochester, where children in more disadvantaged areas have poorer access to better nutritional quality restaurant choices. In London, results suggest an inverse relationship across the city where children in more disadvantaged areas have better access to better nutritional quality restaurant choices. Given these disparate results, research on restaurant nutritional quality for children requires additional consideration.

Childhood obesity, which is related to a number of health issues including type 2 diabetes and CVD, is increasing in Canada and the USA at an alarming rate^(^ ^1^ ^,^ ^2^ ^)^. Obese or overweight children are five times more likely to see obesity and related health issues continue into adulthood^(^ ^3^ ^)^. Surrounding environments and area socio-economic factors play an important role in shaping obesity outcomes^(^ ^4^ ^–^ ^6^ ^)^. Food environments in North America often promote high-energy, high-fat, high-sugar foods and beverages, especially in low-income and highly socio-economically deprived neighbourhoods^(^ ^7^ ^)^. In the USA, over one-third of children consume energy-dense foods daily^(^ ^3^ ^)^, and in Canada over one-quarter of children consume sugar-sweetened beverages daily^(^ ^8^ ^)^. Although American and Canadian cultures are similar in terms of their marketing and availability of unhealthy foods, differences in urban planning policy create differences in the food environment; among other factors, these may ultimately be responsible for the slightly lower rates of obesity in Canada^(^ ^9^ ^,^ ^10^ ^)^.

Because unhealthy foods and drinks are increasingly purchased and consumed by children away from home, it is becoming more necessary to analyse the consumer food environment (FE) as a determinant of diet-related health problems. Glanz *et al.* ^(^ ^4^ ^)^ conceptualized the consumer FE as the price, promotion, placement and availability of healthy options and nutrition information of foods for purchase.* While there has been an increase in consumer FE studies in recent years, the primary focus tends to be on the FE within grocery stores. In a 2014 systematic review of FE for children by Engler-Stringer and colleagues^(^ ^12^ ^)^, only three of twenty-six studies assessed the consumer FE for children, all of which assessed how the FE impacts children’s BMI or diet. None of these studies assessed the content of restaurant children’s menus or their relationship to the surrounding neighbourhood^(^ ^12^ ^)^.

Studies of restaurant consumer FE and the relationship with neighbourhood socio-economic characteristics are increasing on a broad scale. For instance, Larson *et al.* ^(^ ^13^ ^)^ found that restaurants in wealthier areas in the USA offer healthier menu options than low-income areas. This disparity is perhaps unsurprising, as research continuously shows low-income neighbourhoods in the USA have more limited access to healthy foods^(^ ^13^ ^–^ ^15^ ^)^. These neighbourhoods are often referred to as food deserts. Other studies, meanwhile, have found that low-income neighbourhoods in Canada are more likely to be food swamps, having greater access to unhealthy food outlets compared with high-income neighbourhoods^(^ ^14^ ^,^ ^16^ ^)^.

Restaurant consumer FE are often assessed with menu audit tools such as the Nutrition Environment Measures Survey for Restaurants (NEMS-R)^(^ ^17^ ^)^. This tool has been applied in both Canada^(^ ^18^ ^,^ ^19^ ^)^ and the USA^(^ ^20^ ^–^ ^22^ ^)^, but there is rarely a focus on children. To better assess the restaurant consumer FE for children, Krukowski and colleagues created the Children’s Menu Assessment (CMA), a menu audit tool based on the validated NEMS-R that focuses solely on the children’s menu^(^ ^23^ ^)^. The CMA expands on the NEMS-R children’s menu subsection, and is a more comprehensive and extensive means of measuring the FE for children in restaurants^(^ ^23^ ^)^. For example, where the NEMS-R simply asks if a children’s menu has healthy sides available, the CMA has an entire section dedicated to children’s menu sides. This section asks if non-fried vegetables or salads are offered as sides, if fruit is offered as a side and whether it specifies the item is without added sugar, and also asks if dairy products are offered as a side and whether those products specify low-fat^(^ ^23^ ^)^. While the introduction of the CMA has aided in advancing the collective understanding of the consumer FE as it directly pertains to children, its use has been primarily descriptive in nature, falling short of in-depth analysis into how the children’s FE varies based on country context or neighbourhood socio-economic characteristics.

Thus, the purpose of the present study was to assess the restaurant consumer FE for children using a previously unexplored approach: a cross-border comparative analysis of the restaurant consumer FE for children within and between two geographically proximate but internationally distinct North American cities: London, ON, Canada and Rochester, NY, USA. The study’s research objectives are to:

1. determine whether the restaurant consumer FE for children differs between the cities of London, ON, in Canada and Rochester, NY, in the USA; and
2. determine the relationship between neighbourhood restaurant nutritional quality for children and socio-economic characteristics within the city of London, ON and within the city of Rochester, NY.

The study aims to first assess and compare the menu content and nutritional quality (with poor nutritional quality corresponding to low children’s menu scores) in both cities through a descriptive analysis of restaurant children’s menus. The study then examines how the restaurant consumer FE differs within each city and between the two cities using the Neighbourhood Restaurant Quality Index for Children (NRQI-C), described in detail below.

## Methods

### Study setting

The current research was conducted within the city limits of London, ON, Canada and Rochester, NY, USA. London covers 420·35 km^2^, with a population of 383 822 in 2016^(^ ^24^ ^)^, while Rochester is a smaller city covering 96·1 km^2^, with a population of 209 511 as of 2015^(^ ^25^ ^)^. Within London, research has shown exposure to fast, energy-dense food outlets is associated with poor diet quality and increased fast-food purchasing for children and youth – meaning the more exposed a child is to unhealthy options, the more likely s/he is to make unhealthy purchases and have an unhealthy diet^(^ ^26^ ^–^ ^28^ ^)^. The previous research also shows how children actually make choices, whereas the present study builds on that to see what choices are available within restaurants that cater to children. The present study takes what has been previously shown at the individual level (e.g. assessing what choices children are actually making) and expands outwards to examine the FE at the neighbourhood level (e.g. exploring what options are available from which children then make their choices). Although there has been much less research on children’s FE in the City of Rochester, data from the New York State Department of Health show the percentages of overweight and obese children (influenced by dietary behaviours) in Rochester over the last decade have been as high as nearly 50 %^(^ ^29^ ^)^.

Both London and Rochester are located within the Great Lakes region of North America, and both are interior cities nearer to much larger urban regions (Toronto, ON and Buffalo, NY). The two cities also have similar economies, having transitioned from manufacturing to medical sciences and research, although each retains unique strengths in distinctive economic sectors. London and Rochester have also developed under different political and socio-economic conditions, especially with respect to how FE are incorporated into urban planning. FE planning in the USA has been on the rise through the American Planning Association and implementation of the Association’s Policy on Community and Regional Food Planning^(^ ^30^ ^)^. Despite the encouraging attempts that some cities in the USA may have made to incorporate this aspect into their own plans, the official/comprehensive plan of the City of Rochester currently has no mention of food at all. More fundamental to the US context, the laissez-faire or ‘personal responsibility’ approach to American urban planning and decision making generally means a greater disconnect between best practices for health promotion and the way that cities and FE are actually constructed.

Conversely, the Canadian Institute of Planners and the Ontario Professional Planners Institute have both encouraged more discussion around including the FE as a key component of planning^(^ ^31^ ^)^. Similarly, Health Canada has been more involved in examining various aspects of the FE through collaboration with stakeholders^(^ ^16^ ^)^. Unlike the City of Rochester, the official plan of the City of London includes 120 mentions of the word ‘food’ and addresses how the city will meet goals related to the FE in the future, including ensuring all Londoners have access to food sources providing affordable, safe, healthy, local foods^(^ ^32^ ^)^. In this sense, the Canadian style of planning and FE policy is much more aligned with the idea that government should play a role in the delivery of healthy environments. By using two study areas in contrasting locations, the present paper aims to build on the underdeveloped knowledge base examining Canada–US differences in the consumer FE for children.

### Restaurant assessment

We obtained the addresses for fast-food and full-service restaurants from the City of Rochester Planning Department and the Middlesex-London Health Unit. Address locations were geocoded in a geographic information system (ArcGIS version 10.3) and verified through websites, Google Maps and Street View, phone calls and site visits. Multiple types of restaurants were included rather than focusing solely on fast-food restaurants because full-service restaurants typically offer the same items as their fast-food counterparts. Because of this, assessing fast-food restaurants as the sole source of unhealthy restaurant entrées and options would vastly underestimate neighbourhood exposure to unhealthy foods in the restaurant consumer FE^(^ ^33^ ^)^.

After verifying the addresses, all existing children’s menus were collected within each study area between June and August 2016. The online menu of each restaurant in the study area was consulted and saved if the children’s menu was posted. If the online menu did not include a children’s menu, a phone call was made to the restaurant to confirm whether the restaurant offered a children’s menu in-store. Restaurants confirmed as offering children’s menus in-store were then visited in-person for collection.

Each children’s menu was assessed and scored using the CMA tool, which consists of questions regarding healthfulness of main dishes, proportion of whole to white grains, desserts, beverages, sides, nutritional information, toy promotions and branded marketing^(^ ^23^ ^)^. Total CMA score ranges from −5 to 21, where higher scores correspond to greater availability or greater menu presence of healthy choices^(^ ^23^ ^)^. Previous CMA studies do not divide menu scores into various nutritional quality categories. Based on the natural breaks in the menu scores, poor nutritional quality menus were categorized as those menus with CMA scores of 0 or lower, average nutritional quality menus as those with CMA scores from 1 to 4, and high nutritional quality menus as those with CMA scores of 5 or higher.

The term ‘healthy’ is used throughout the present paper to describe menu items and is based on the definitions and instructions listed on the CMA, which puts the burden of proof on the restaurant, rather than the researcher scoring the menu, to identify whether items are healthy or not. The restaurant menu must explicitly state that menu items are prepared in a healthy way or that beverages are explicitly 100 % fruit juice or low-fat milk. If the menu does not state this, the menu is scored accordingly. For example, the CMA instructs that a main dish prepared as grilled, baked, smoked or broiled would be considered healthy when referring to proteins such as chicken or fish, while a sandwich that is grilled, such as grilled cheese, is not necessarily healthy even though it is described as grilled^(^ ^23^ ^)^. Two raters independently assessed and scored each children’s menu in the study area and when discrepancies arose, a third rater was consulted. Percentage agreement was then estimated by dividing the number of children’s menus with scores in agreement by both raters by the total number of children’s menus included. This value was then converted to a percentage. Inter-rater reliability for this study was high, as mean percentage agreement was 94·6 %.

### Quantifying restaurant accessibility/opportunity

To make the children’s menu score more meaningful at the neighbourhood level, a new Neighbourhood Restaurant Quality Index for Children (NRQI-C) was created. This novel index represents restaurant accessibility/opportunity measures from each residential neighbourhood. The NRQI-C is best calculated at the block level (census block in the USA or dissemination block in Canada), as this allows for a finer understanding of local-level variations in accessibility to restaurants and fast-food outlets, and is calculated as follows:

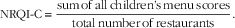

Restaurants with a children’s menu were assigned the respective menu score calculated from the CMA. Restaurants that did not offer a children’s menu were assigned a score of 0. Using the Network Analyst extension of ArcGIS version 10.3, 800 m network service areas or network buffers were created from the centroid of each block. Network buffers, which are zones of a defined 800 m radius calculated along the street network, were used as they more accurately depict the area that influences walking, whereas circular buffers (i.e. Euclidean buffer distances measured ‘as the crow flies’) are more likely to ignore barriers to walking (e.g. private land, rivers and/or railroads that are difficult to cross)^(^ ^34^ ^)^. As well, restaurants are destinations, and employing a network buffer around the block centroid better encapsulates the variety of restaurants around a neighbourhood. Without this network buffer step, a restaurant just outside the block may be missed, resulting in an edge effect and inaccurate results^(^ ^35^ ^)^. The buffer distance of 800 m was chosen as it is commonly used among food access studies^(^ ^36^ ^,^ ^37^ ^)^ and among children’s FE studies^(^ ^12^ ^)^, and is a distance often recognized as walkable in 10 to 15 min^(^ ^6^ ^)^.

After calculating the network buffers, the spatial join function was employed to determine the total number of restaurants and the sum of the children’s menu scores within each buffer. With these two values, NRQI-C was then calculated and assigned to the buffer’s respective block. This process was repeated for every block within the city limits for Rochester and London. To account for un- or sparsely populated census blocks, NRQI-C scores were weighted by population. This was done by dividing the block population by the corresponding block group (BG) or dissemination area (DA) population and multiplying that value by the NRQI-C for the block. This process ensures that the NRQI-C score accurately reflects the population of the respective area. Because the smallest level at which demographic census data is released is the BG (in the USA) and DA (in Canada) – one level up from block – the average weighted NRQI-C of all blocks was then calculated in the corresponding BG or DA using the summarize table tool.

### Correlation and regression models

Once each BG or DA was assigned its corresponding NRQI-C, we ran Pearson correlation coefficients and multiple regression models to assess the relationship between neighbourhood restaurant nutritional quality for children and variables of the socio-economic environment including population density, density of children (0–14 years), median household income, percentage of income from public assistance (in Canada, this is referred to as government transfer payments), percentage of families headed by lone parents and percentage of the population identifying as a visible minority. In Canada, a visible minority is anyone who identifies as a race/ethnicity other than Indigenous or Caucasian/white^(^ ^24^ ^)^, so for sake of comparison we created a similar variable for Rochester which included those who identified as a race/ethnicity other than Native American or Caucasian/white. Additionally, we conducted separate regression analyses for each city, Rochester and London, using NRQI-C as the outcome variable. In each regression, we retained only the independent variables that the bivariate correlations revealed were significantly correlated with NRQI-C (*P*≤0·05 was deemed statistically significant).

## Results

Our original list included 926 restaurants within London city limits, with 323 (34·9 %) identified as having separate children’s menus, and 242 restaurants within Rochester city limits, with fifty (20·7 %) identified as having separate children’s menus.

### Children’s Menu Assessment descriptive results

Total menu scores for all fifty menus in Rochester ranged from −2 to 13, with a mean score of 3·12 (sd 3·69). Total menu scores for all 323 menus in London ranged from −3 to 9, with a mean score of 2·87 (sd 3·09). These total scores are described in Table 1. Menus with poor nutritional quality were defined as those scoring 0 or lower. In Rochester, twenty-one menus (42·0 %) scored 0 or lower, ten (20·0 %) had a score between 1 and 4, and nineteen (38·0 %) had a score of 5 or higher. In London, ninety-three menus (28·8 %) scored 0 or lower, 128 (39·6 %) scored between 1 and 4, and 102 (31·6 %) had a score of 5 or higher. This suggests that compared with London, Rochester has a greater proportion of children’s menus with better nutritional quality (score of 5 or higher), as well as a greater proportion with poorer nutritional quality (score of 0 or lower).

**Table 1:** Number of menus that received a score, within each category of Children’s Menu Assessment (CMA) score, in the cross-border comparative analysis of the restaurant consumer food environment for children in London, ON, Canada and Rochester, NY, USA (June–August 2016)

	Rochester (*n* 50)		London (*n* 323)	
Total CMA score	*n*	%	*n*	%
0 or lower	21	42·0	93	28·8
1 to 4	10	20·0	128	39·6
5 or higher	19	38·0	102	31·6
9 or higher	8	16·0	4	1·24

In Rochester, significantly more children’s menus included a toy in the children’s meal than London (20·0 *v*. 9·6 %, *P*=0·05) and used branded marketing as a means of promotion (20·0 *v*. 7·1 %, *P*=0·01). Children’s menus in London were more likely to use symbols to indicate healthy items compared with Rochester (11·1 *v*. 2·0 %, *P*=0·04). London had a higher proportion of children’s menus that automatically included unhealthy desserts (e.g. ice cream) with meals than Rochester (38·4 *v*. 10·0 %, *P*<0·001), and London was also more likely to offer pop (45·2 *v*. 24·0 %, *P*=0·01). Very few children’s menus in either city offered healthy desserts (e.g. fresh fruit; i.e. 0 % in Rochester, 5·9 % in London, *P*=0·06). In London, 31·9 % of children’s menus offered at least one healthy main dish, while 26·0 % in Rochester had at least one healthy main dish. Similarly, more London menus (26·0 %) offered a non-fried vegetable side such as a salad or steamed broccoli compared with Rochester (16·0 %), although the London–Rochester differences regarding healthy main dishes and sides were not significant. Additional results and the comparison between the two cities can be found in Table 2, which breaks down each CMA scoring category and identifies the number and percentage of menus in each city found to have met the criteria.

**Table 2:** Children’s Menu Assessment (CMA) categories scored in the cross-border comparative analysis of the restaurant consumer food environment for children in London, ON, Canada and Rochester, NY, USA (June–August 2016)

	Rochester (*n* 50)			London (*n* 323)			
CMA category	*n*	%	Mean score	*n*	%	Mean score	*P* value
Nutrition guidance							
Any nutrition information	15	30·0	0·60	91	28·2	0·57	0·92
Symbol indicating healthy item	1	2·0	0·02	36	11·1	0·11	0·04
Entrées							
Healthy entrée	13	26·0	0·48	103	31·9	0·55	0·50
Healthy entrée salad	2	4·0	0·08	8	2·5	0·05	0·63
Whole-grain option	7	14·0	0·28	64	19·8	0·25	0·44
Beverages							
Juice, any	22	44·0	N/A	154	47·7	N/A	0·74
Juice, listed as 100 % juice	7	14·0	0·14	27	8·4	0·08	0·31
Milk, any	24	48·0	N/A	172	53·3	N/A	0·59
Milk, listed as low-fat, 1 %, or non-fat	10	20·0	0·20	37	11·5	0·11	0·14
Pop targeted at children	12	24·0	−0·20	146	45·2	−0·45	0·01
Opportunity for healthier beverage substitution	27	54·0	0·54	181	56·0	0·56	0·91
Free pop refills for children	1	2·0	−0·02	25	7·7	−0·08	0·23
Side dishes							
Non-fried vegetables	8	16·0	0·32	84	26·0	0·52	0·18
Fruit, any	23	46·0	0·46	113	35·0	0·35	0·18
Fruit, without added sugar	10	20·0	0·20	90	27·9	0·28	0·32
Dairy, any	2	4·0	0·04	12	3·7	0·04	1·00
Dairy, low-fat	0	0·0	0·00	0	0·0	0·00	N/A
Opportunity for healthier side substitution	24	48·0	0·48	171	52·9	0·53	0·62
Desserts							
Healthy desserts	0	0·0	0·00	19	5·9	0·06	N/A
Included in children's meal	5	10·0	−0·10	124	38·4	−0·39	<0·001
Toys/marketing							
Branded marketing towards children	10	20·0	−0·20	23	7·1	−0·02	0·01
Toy included with children's meal	10	20·0	−0·20	31	9·6	−0·07	0·05
Total score	N/A	N/A	3·12	N/A	N/A	2·87	N/A

N/A, not applicable. The *χ* ^2^ test, or Fisher’s exact test where appropriate, was used to compare differences in proportions between Rochester and London.

### Correlation and regression analyses of Neighbourhood Restaurant Quality Index for Children data

**Fig. 1:**
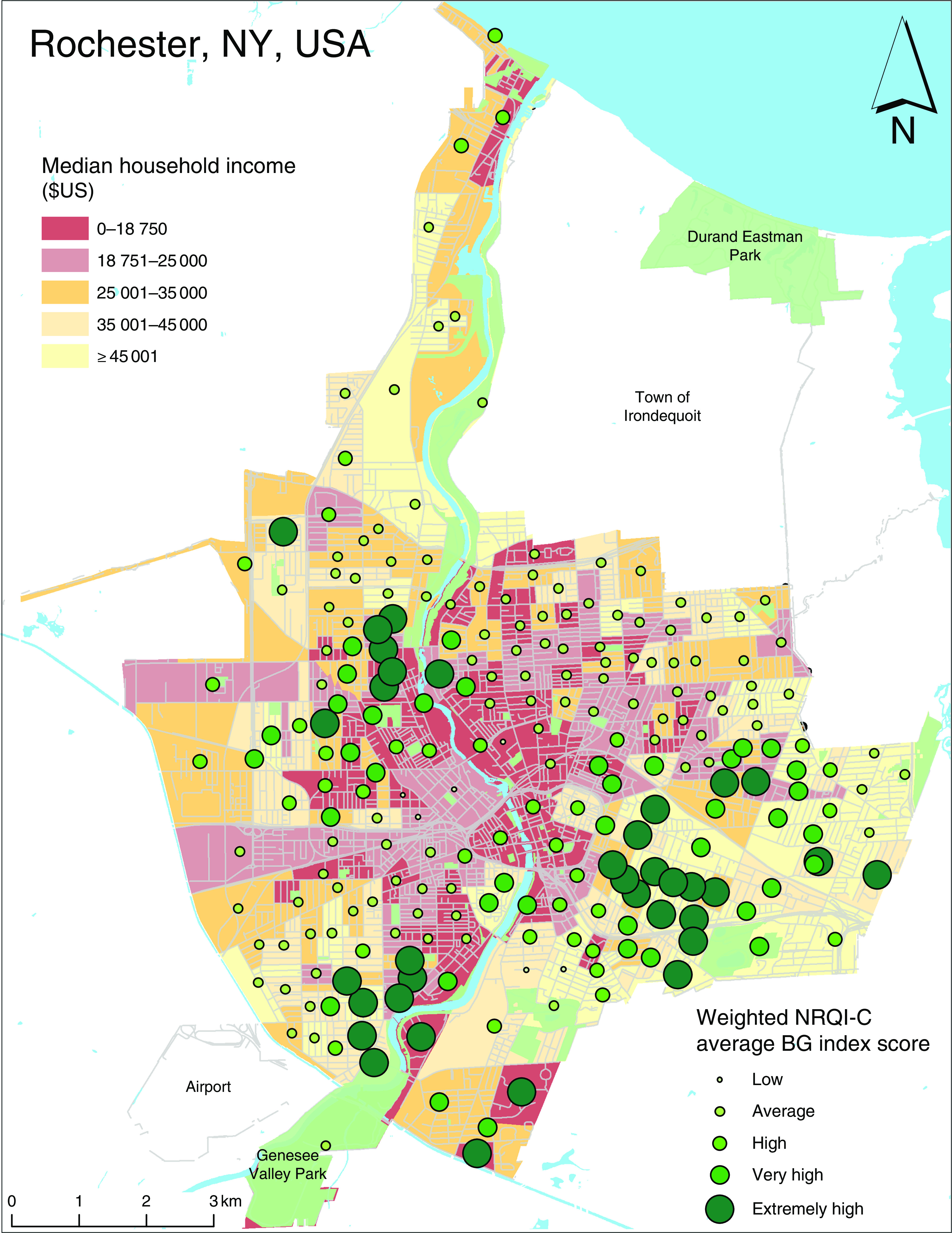
(colour online) Relationship between the Neighbourhood Restaurant Quality Index for Children (NRQI-C) weighted by population and median household income in Rochester, NY, USA (June–August 2016), per census block group (BG)

In Rochester, Pearson’s correlation analysis (Table 3) revealed that higher NRQI-C scores were positively correlated with higher median household income (*r*=0·14, *P*<0·05), suggesting the nutritional quality of menus increases as neighbourhood median household income increases. This association can be seen in Fig. 1, where the higher-income (lighter coloured) BG have higher NRQI-C values. Additionally, NRQI-C scores were also negatively correlated with density of children (*r*=−0·15, *P*<0·05) and per cent visible minority (*r*=−0·18, *P*<0·01; Table 3). This suggests that menu nutritional quality is poorer in neighbourhoods where there is a greater concentration of children or a larger proportion of the neighbourhood population is a visible minority. The multiple regression analysis for Rochester (Table 4) revealed that only the proportion of visible minorities remained significantly associated with NRQI-C scores after adjusting for household income and density of children, such that a higher proportion of visible minorities was associated with lower NRQI-C scores (*P*=0·01).

**Table 3:** Correlations between Neighbourhood Restaurant Quality Index for Children (NRQI-C) score and variables of the social environment in the cross-border comparative analysis of the restaurant consumer food environment for children in London, ON, Canada and Rochester, NY, USA (June–August 2016)

Socio-economic status variable	Rochester	London
Population density	−0·01	0·01
Density of children	−0·15*	0·07
Median household income	0·14*	−0·13**
Public assistance (%)	−0·09	0·02
Lone parenthood (%)	−0·05	0·15***
Visible minority (%)	−0·18**	0·11**

The Pearson correlation coefficient was used to measure the association between NRQI-C and each socio-economic status variable. *P<0·05, **P<0·01, ***P<0·001.

**Table 4:** Regression of Neighbourhood Restaurant Quality Index for Children (NRQI-C) score *v*. median household income, visible minority (%) and density of children in Rochester, NY, USA (June–August 2016)

	*β*	*t*	*P*	Adjusted *R* ^2^
				0·048
Median household income	0·003	0·036	0·971	
Visible minority (%)	−0·244	−2·575	0·011	
Density of children	−0·001	−0·012	0·990	

**Fig. 2:**
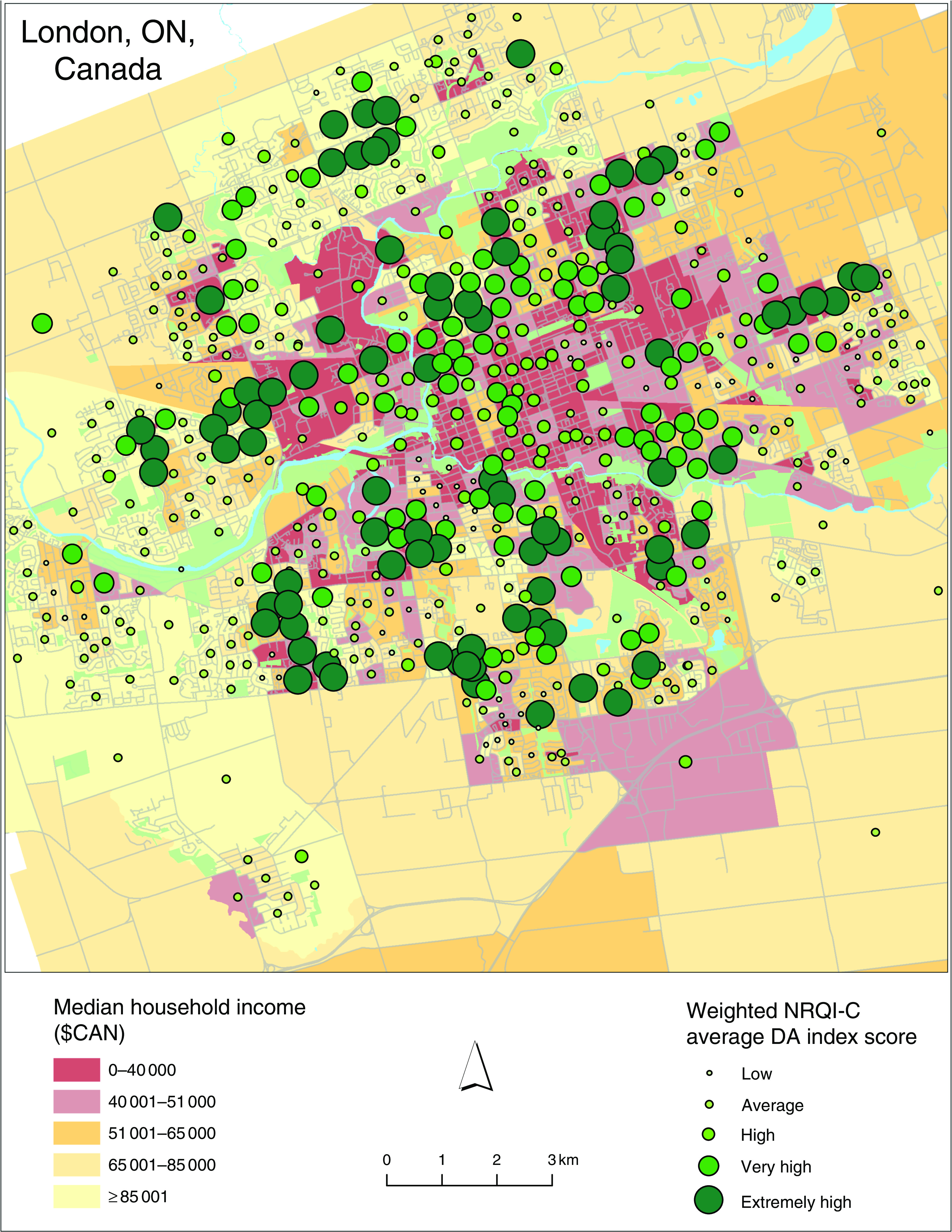
(colour online) Relationship between the Neighbourhood Restaurant Quality Index for Children (NRQI-C) weighted by population and median household income in London, ON, Canada (June–August 2016), per census dissemination area (DA)

In London, by contrast, Pearson’s correlation analysis (Table 3) revealed that higher NRQI-C scores were negatively correlated with median household income (*r*=−0·13, *P*<0·01), but positively associated with per cent visible minority (*r*=0·11, *P*<0·01) and percentage of families headed by lone parents (*r*=0·15, *P*<0·001). These results suggest that the nutritional quality of menus is higher in neighbourhoods with a high percentage of visible minorities and lone-parent families, but lower in areas of high household income (Fig. 2). The multiple regression revealed that only per cent visible minority remained a significant predictor of NRQI-C in London (Table 5); NRQI-C scores were higher in neighbourhoods with a higher proportion of visible minority residents (*P*=0·026), which was opposite in Rochester.

**Table 5:** Regression of Neighbourhood Restaurant Quality Index for Children (NRQI-C) score *v*. median household income, lone parenthood (%) and visible minority (%) in London, ON, Canada (June–August 2016)

	*β*	*t*	*P*	Adjusted *R* ^2^
				0·027
Median household income	−0·041	−0·709	0·478	
Lone parenthood (%)	0·112	1·958	0·051	
Visible minority (%)	0·093	2·239	0·026	

## Discussion

To our knowledge, the present study is the first to investigate the children’s restaurant FE in two cities on either side of the Canada–US border, and the first to develop and implement an index for neighbourhood restaurant quality in children (NRQI-C). Several studies have employed the CMA in various settings to examine the restaurant FE for children^(^ ^11^ ^,^ ^22^ ^,^ ^38^ ^–^ ^43^ ^)^, but virtually none go beyond descriptive results to analyse the relationship between FE and neighbourhood socio-economic characteristics. An exception is Hill *et al.* ^(^ ^38^ ^)^, who explored how children’s menu scores differ in urban and rural areas, as well as by block group race/ethnicity. A second recent exception is DuBreck *et al.* ^(^ ^11^ ^)^, who examined children’s menu scores in urban, suburban and rural areas, and how the menu items vary geographically as well as based on socio-economic status. Our study reports on the findings of the children’s menu audits in two urban study areas and expands to also consider variations between representative American and Canadian cities.

In Rochester, we discovered a weak, but positive relationship between NRQI-C score and median household income such that as neighbourhood income increases, more restaurants not only offer a children’s menu, but also healthier options. This is consistent with FE literature in the USA which suggests those in higher-income neighbourhoods have better access to healthier foods than those in lower-income neighbourhoods^(^ ^13^ ^–^ ^15^ ^)^. Regression analysis, however, revealed that the only significant predictor of neighbourhood NRQI-C in Rochester was per cent visible minority, such that a larger presence of visible minorities was associated with poorer NRQI-C score. This suggests that neighbourhoods with more minorities will have fewer restaurants offering children’s menus, and if a children’s menu does exist, the nutritional quality will be substantially poorer in than a neighbourhood with a lower proportion of visible minorities. These results highlight the socio-economic inequalities characterized by US FE studies and are again consistent with the structural differences characterized by planning in the USA, which favours private sector-led development (i.e. restaurants). This can lead to landscapes less protective of public health despite the paradoxical fact that modern zoning as a means of protecting public health was born in the USA^(^ ^44^ ^)^. The results are also consistent with FE literature in the USA that suggests high-minority neighbourhoods have poorer access to healthier options^(^ ^45^ ^)^.

In London, higher NRQI-C score was associated with lower median household income, suggesting that as neighbourhood income decreases, more restaurants have children’s menus and healthier options. One reason for this may be that many of the highest scoring children’s menus were those found in fast-food chains (e.g. McDonald’s, Wendy’s, Burger King, Subway). These chain locations in Canada – although stereotyped as and predominantly still unhealthy – have started offering more non-fried sides (e.g. yoghurt is automatically included as a side for some chains), healthier drink options (e.g. bottled water is promoted as a beverage choice on menus), healthier main dish alternatives (e.g. grilled chicken wraps are promoted as a main dish choice on menus), and display nutritional information for children. Although these chains are also found in the USA, not all these healthier menu choices have been observed in the USA. Research in Canada suggests lower-income neighbourhoods have greater access to outlets such as fast-food restaurants^(^ ^14^ ^,^ ^16^ ^)^ and since many of these restaurants had high scoring (healthy) children’s menus, the NRQI-C was higher in those areas in London.

We also found that higher NRQI-C score was associated with higher percentage of lone-parent families in London, suggesting neighbourhoods with higher percentages of lone-parent families have a higher quantity and nutritional quality of children’s menus. This aligns with the City of London’s official plan, which aims to provide all Londoners, and in this case London families that may not have two parents available to shop for food, with access to affordable, safe, healthy, local foods^(^ ^32^ ^)^. Meanwhile, regression analysis for London revealed that the only significant independent variable that was associated with NRQI-C was per cent visible minority. In this case, the relationship was opposite to what was found in Rochester; in London, higher NRQI-C scores were found in neighbourhoods with a greater proportion of visible minority residents. It is likely that this relationship between NRQI-C and per cent visible minority is positive in London because the presence of racial segregation and limited opportunities is not as prominent as in US cities such as Rochester. In London, some of the high-visible-minority areas are also high-income areas, so the stigmatization and barrier to healthy food access are not as apparent. Some literature suggests the ‘Americanization’ of recent immigrants – where weight is rapidly gained after arriving to the USA – as American-type food is seen as a status symbol and a way to acclimatize to North American culture^(^ ^46^ ^,^ ^47^ ^)^.

Despite the size difference between the two cities and the subsequent number of children’s menus assessed, the contents of children’s menus tended to be similar between Rochester and London (Table 2). Rochester, however, had a significantly lower percentage of menus offering pop specifically targeted towards children and unhealthy desserts included in a children’s meal. This may be because Rochester has several restaurants within the city limits that push eating healthy as part of the region’s ‘5-2-1-0 Be a Healthy Hero’ initiative and the ‘Healthy Hero Restaurant’ programme. These initiatives within the Rochester area are similar to initiatives in other communities across the USA and encourage children to engage in healthier activities every day because one in three children in the city and surrounding area is overweight or obese^(^ ^48^ ^)^. The highest scoring children’s menu (CMA total score=13) was even titled ‘Healthy Hero Menu Choices’ and included items such as grilled turkey, fresh fruit and broccoli – providing healthy options from which children can choose.

A similar children’s healthy lifestyle initiative in the City of London is called the Healthy Kids Community Challenge (HKCC), a three-part province-wide programme focusing on encouraging children to engage more in physical activity, drink more water, and eat more fruits and vegetables. Interestingly, although programme promotion exists within the city, none of the children’s menus assessed in London advocated for the HKCC. Nevertheless, promotion in London was significantly different from Rochester in that a greater percentage of its children’s menus used symbols to indicate healthy items and a significantly smaller percentage included toys and used branded marketing targeting children. Despite faring better in this area than Rochester, the uptake of healthy marketing on children’s menus in London is still very small, and there is a clear opportunity for public health and government officials to use the results of the present study to engage in conversations and interventions with local restaurants to create and promote healthier menus for children.

The findings of the present study demonstrate how opposite relationships exist between children’s menu nutritional quality and neighbourhood socio-economic characteristics in different contexts (in this case, London, ON and Rochester, NY). In London, we discovered that higher scoring children’s menus (better nutritional quality foods) were associated with areas of higher percentage of visible minority residents. The relationship in Rochester, however, was the opposite; higher scoring children’s menus were found in areas with a lower percentage of visible minorities. We expect such differences would persist to some degree in other comparative cross-border studies (given fundamental differences in planning policy between Canadian and US cities) and therefore emphasize the need for additional cross-border inquiries using similar methods.

### Policy implications

Car-centric land-use policy and nutrition policy focused on large-scale agri-food system interests have driven inequalities in exposure to unhealthy foods, including via the proliferation of big box supermarkets and chain fast-food restaurants^(^ ^49^ ^,^ ^50^ ^)^. Despite cross-border differences in land-use planning, exposure to unhealthy foods exists in both countries, suggesting a continued need for a focus on food system policy. The focus of these policies on agri-food interests has likely contributed to the health problems associated with consumption of unhealthy food products in both countries^(^ ^51^ ^–^ ^54^ ^)^. In the USA, former President Barack Obama and First Lady Michelle Obama’s efforts toward improving FE for children through the ‘Let’s Move!’ campaign may be responsible for improving some of the cross-border disparity in restaurant menu nutritional quality^(^ ^55^ ^)^. Given continuing diet-related health issues, however, it is troubling that a public health focus on policy making remains largely absent from food system policy^(^ ^56^ ^–^ ^58^ ^)^.

A direct application of the current research is to use this evidence to encourage changes to the consumer FE; that is, to what foods can be sold or marketed to children in restaurants. Indeed, some large chain restaurants have already begun offering healthier options due to consumer pressure^(^ ^59^ ^)^, and continued pressure may help further effect change. As noted below in more detail, other policy levers include introducing new taxes to discourage the creation of unhealthy food items. Although changing FE in restaurants is necessary and directly tied to the present research, the inequalities we found geographically also provide a tool for advocating for healthier community FE.

With mounting evidence that disparities in exposure to unhealthy foods are rooted in modifiable land-use patterns – including in the present paper and in past work in the study site of London^(^ ^28^ ^,^ ^60^ ^)^ – public health practitioners on both sides of the border would be well served by increasing their advocacy around this topic as local-level advocates have the capacity to effect change. Such built environment changes could include restrictions on siting of fast-food restaurants, enacting sign ordinances to limit the size of advertising, and promoting healthy environments in ways not directly tied to but that counteract the negative effects of the food system (such as through safe and active living).

The present study shines a light on how food marketed to children needs to be changed and provides concise figures that policy makers can use to intervene, whether in a localized area or on a local, provincial/state or federal level. Public health and governmental officials can use these results to improve relationships with restaurants and to encourage the inclusion of healthy menu choices for children, as well as use these results to tailor future interventions to focus on restaurant children’s menus. These types of interventions involve the public sector intervening in the private sector, but have been done before. Many children’s menus offer soda/pop as the default beverage with an entrée and charge an additional fee for beverages such as milk or juice.

Similarly, many menus offer healthy sides such as salads or vegetables but at an additional cost, whereas fried sides are included in the price. Policy makers could use the results presented here to target restaurants and place a tax on these unhealthy beverages and sides. Many countries have taxes on sugary drinks and foods with high energy density, although not specifically targeting restaurant children’s menus^(^ ^61^ ^–^ ^65^ ^)^. Both federal and provincial/state policy makers can create and enforce taxes like this, or ban unlimited refills of pop and other sugar-sweetened beverages altogether as has been done elsewhere^(^ ^65^ ^)^. In the meantime, researchers should continue to employ the CMA in other cities in order to build a thorough and collective understanding of restaurant consumer FE for children.

### Limitations

Although we included all restaurants in the NRQI-C analysis regardless of children’s menu presence, only children’s menus were assessed and scored using the CMA. We acknowledge that children often do order off the general menu and we recommend menu assessments be conducted in the future on all restaurant menus in both study areas. As well, although the CMA is based on the validated NEMS-R tool, studies have not as yet validated the CMA itself. This is a potentially valuable contribution future studies could make to the literature.

The results from the analysis conducted using the newly created NRQI-C are consistent with previous FE literature. Since this is the first time it has been implemented, however, no other studies exist with which results can be compared. Thus, future studies should incorporate the use of the NRQI-C to measure the neighbourhood FE.

Additionally, the present research examines neighbourhood restaurant nutritional quality for children through a walkable 800 m area within individual neighbourhoods. We recognize, however, that mobility does play a role in food access and encourage future studies to build on this research to examine the NRQI-C within a larger, drivable distance to see how the index values may vary or how aggregate estimates derived from activity spaces may relate to consumption.

Several restaurants called during the data collection period indicated there was no physical children’s menu, but that the establishment served ‘kid-friendly items’ or offered child-sized portions of main dishes on request. Because there was no physical children’s menu, these restaurants were excluded from the study as the CMA assesses the separate children’s menu only.

Although the cities of London and Rochester are comparable in many ways, London is much larger in size than Rochester and its city limits incorporate urban, suburban and rural areas. The city limits of Rochester encompass an urban area only and the nearby suburban and rural areas are separate municipalities. Because of this, there is a large difference in menu sample size between these two study sites. Future studies may build on this to compare regions, rather than confine the study areas to within city boundaries.

Finally, the present research highlights what choices are available to children in restaurants but does not examine what items are ordered from these menus. Future studies may build on this research to explore how menu choices are made (e.g. between parent and child) and what menu choices are ordered within the restaurant consumer FE, as well as to understand how satisfied or dissatisfied parents and children are with an increased presence of healthier menu options.

## Conclusion

Childhood obesity-related health issues are on the rise and research suggests the rise is linked with dietary behaviours. Several studies examine the nutritional quality of restaurant children’s menus specifically using the CMA, but none have applied the tool to compare cities in two different countries or incorporated the use of a child-focused restaurant nutritional quality index. The novel approach of the present study is useful in highlighting the variety of categories that exist on children’s menus that warrant further research both within and between cities. The study adds to the consumer FE literature for children, specifically within inner-city neighbourhoods, and augments our understanding of the nutritional quality and options available among North American children’s menus. The study builds on previous research on children’s menus in Southwestern Ontario and is the first study to employ the CMA in Rochester, NY, as well as the first to compare children’s menus in two countries, across their common border. Children’s menus are rarely the focal point of consumer FE research, but there is still much to be learned, and much to be done, as childhood obesity rates – influenced by poor dietary habits – continue to rise on a global scale.

## Footnotes

* These are in contrast to measurements of the community FE, which typically estimate the number or density of stores within certain distances from homes, schools or activity spaces^(^ ^11^ ^)^.
